# A Sharp Threshold Phenomenon in String Graphs

**DOI:** 10.1007/s00454-021-00279-3

**Published:** 2021-03-01

**Authors:** István Tomon

**Affiliations:** ETH Zürich, Zürich, Switzerland

**Keywords:** String graph, Separator, 05C10

## Abstract

A string graph is the intersection graph of curves in the plane. We prove that for every $\epsilon >0$, if *G* is a string graph with *n* vertices such that the edge density of *G* is below ${1}/{4}-\epsilon$, then *V*(*G*) contains two linear sized subsets *A* and *B* with no edges between them. The constant 1/4 is a sharp threshold for this phenomenon as there are string graphs with edge density less than ${1}/{4}+\epsilon$ such that there is an edge connecting any two logarithmic sized subsets of the vertices. The existence of linear sized sets *A* and *B* with no edges between them in sufficiently sparse string graphs is a direct consequence of a recent result of Lee about separators. Our main theorem finds the largest possible density for which this still holds. In the special case when the curves are *x*-monotone, the same result was proved by Pach and the author of this paper, who also proposed the conjecture for the general case.

## Introduction

The *intersection graph* of a family of sets $\mathscr {C}$ is the graph whose vertices are identified with the elements of $\mathscr {C}$, and two vertices are joined by an edge if the corresponding elements of $\mathscr {C}$ have a non-empty intersection. A *curve* in the plane is the image of a continuous function $\phi :[0,1]\rightarrow \mathbb {R}^{2}$, and a *string graph* is the intersection graph of a family of curves.

Combinatorial and computational properties of string graphs are extensively studied, from both a theoretical and practical points of view. The concept of string graphs was introduced by Benzer [1] in order to study topological properties of genetic structures. Later, Sinden [18] considered string graphs to model printed circuits, and he proved the already non-trivial statement that not every graph is a string graph.

A *separator* in a graph *G* is a subset *S* of the vertices of *G* such that every connected component of $G-S$ has size at most ${2|V(G)|}/{3}$. A classical result of Lipton and Tarjan [13] is that every planar graph on *n* vertices contains a separator of size $O(\sqrt{n})$. Building on this result, Fox and Pach [6] proved that if $\mathscr {C}$ is a family of curves and *m* is the total number of crossings between the elements of $\mathscr {C}$, then the intersection graph of *G* contains a separator of size $O(\sqrt{m})$. They conjectured a strengthening of their result that the same conclusion holds if *m* denotes the number of intersecting pairs of curves in $\mathscr {C}$. Almost settling this conjecture, Matoušek [14] proved that every string graph *G* with *m* edges contains a separator of size $O(\sqrt{m}\log m)$. Recently, the conjecture of Fox and Pach was confirmed by Lee [12]:

### Theorem 1.1

If *G* is a string graph with *m* edges, then *G* contains a separator of size $O(\sqrt{m})$.

Fox and Pach [6, 8] gave several applications of the existence of small separators in string graphs.

**Fig. 1:**
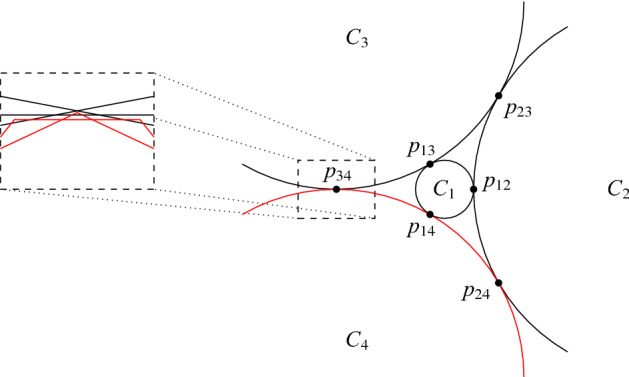
Consider four pairwise touching circles $C_{1},C_{2},C_{3},C_{4}$ that touch at the six points $p_{ij}$ for $1\le i<j\le 4$. For each vertex in $V_{i}$, we can define a convex set that only slightly deviates from the circle $C_{i}$. It is possible to define these convex sets so that if $x\in V_{i}$ and $y\in V_{j}$ for some $1\le i<j\le 4$, then the corresponding convex sets intersect in a small neighborhood of $p_{ij}$ if $xy\in E(G)$, and these convex sets are disjoint otherwise

A *bi-clique* in a graph *G* is a pair of disjoint subsets of vertices (*A*, *B*) such that $|A|=|B|$ and $ab\in E(G)$ for every $a\in A$ and $b\in B$, and the *size* of a bi-clique (*A*, *B*) is |*A*|. An immediate consequence of Theorem 1.1 is that for every $\delta$, $0<\delta <{1}/{4}$, there exists $c>0$ such that if *G* is a string graph with *n* vertices and at most $cn^{2}$ edges, then $\overline{G}$ (the complement of *G*) contains a bi-clique of size at least $\delta n$. Pach and Tomon [16] proved that if we restrict our attention to *x-monotone curves* (curves that intersect every vertical line in at most one point), then there is a sharp threshold for the edge density when linear sized bi-cliques start to appear in $\overline{G}$. More precisely, they proved that for every $\epsilon >0$ there exists $\delta >0$ such that if *G* is the intersection graph of *n* *x*-monotone curves and $|E(G)|\le ({1}/{4}-\epsilon ){n^{2}}/{2}$, then $\overline{G}$ contains a bi-clique of size at least $\delta n$. On the other hand, they showed that there exists a family of *n* convex sets^1^ whose intersection graph *G* has at most $({1}/{4}+\epsilon ){n^{2}}/{2}$ edges, but the size of the largest bi-clique in $\overline{G}$ is $O(\epsilon ^{-1}\log n)$. Indeed, the existence of such families is a consequence of the following result of Pach and Tóth [17]: if *G* is a graph whose vertex set can be partitioned into four parts $V_{1},V_{2},V_{3},V_{4}$ such that $V_{i}$ induces a clique for $i=1,2,3,4$, then *G* can be realized as the intersection graph of convex sets. See Fig. 1 for the basic idea of the construction. But then, a standard probabilistic construction shows that there exist such graphs *G* with *n* vertices, at most $({1}/{4}+\epsilon ){n^{2}}/{2}$ edges, and such that the size of the largest bi-clique in $\overline{G}$ is $O(\epsilon ^{-1}\log n)$. Interestingly (but not surprisingly!), a similar argument shows that the total number of string graphs on *n* vertices is at least $2^{({3}/{4}+o(1)){n^{2}}/{2}}$, which is sharp up to the error term, see the recent result of Pach et al. [15] about the structure of typical string graphs.

Pach and Tomon [16] also conjectured that their result holds without the assumption that the curves are *x*-monotone. Our main result is the proof of this conjecture.

### Theorem 1.2

For every $\epsilon >0$ there exists $\delta >0$ such that the following holds. For every positive integer *n*, if *G* is a string graph with *n* vertices and at most $({1}/{4}-\epsilon ){n^{2}}/{2}$ edges, then there exist two disjoint sets $A,B\subset V(G)$ such that $|A|=|B|\ge \delta n$ and there are no edges between *A* and *B*.

Recently, a great deal of research was focused on finding large bi-cliques in intersection graphs and in their complements, due to their connection to Erdős–Hajnal type questions [5]. Fox et al. [9] proved that if *G* is the intersection graph of *n* convex sets, then *G* or its complement contains a bi-clique of size $\Omega (n)$. Also, if *G* is the intersection graph of *n* *x*-monotone curves, then *G* contains a bi-clique of size $\Omega ({n}/{\log n})$, or $\overline{G}$ contains a bi-clique of size $\Omega (n)$, and these bounds are best possible. Pach and Tomon [16] gave a different proof of the latter result using ordered graphs. Fox et al. [10] also proved that for every $k\in \mathbb {N}$ there exists $c_{k}>0$ such that if *G* is the intersection graph of *n* curves, where any two of the curves cross at most *k* times, then *G* or its complement contains a bi-clique of size at least $c_{k}n$.

When there are no restriction on the curves, Fox and Pach [7] proved that every dense string graph contains a dense incomparability graph as a subgraph. By results of Fox [4] and Fox et al. [9], this implies that every dense string graph contains a bi-clique of size $\Omega ({n}/{\log n})$. Combining this with the result of Lee [12], we get that if *G* is a string graph, either *G* contains a bi-clique of size $\Omega ({n}/{\log n})$, or $\overline{G}$ contains a bi-clique of size $\Omega (n)$; again, these bounds are best possible. Let us remark that incomparability graphs exhibit a similar threshold phenomenon as Theorem 1.2. Indeed, if *G* is an incomparability graph with *n* vertices and at most $({1}/{2}-\epsilon ){n^{2}}/{2}$ edges, then $\overline{G}$ contains a bi-clique of size $\Omega (\epsilon n)$, but there are incomparability graphs *G* with at most $({1}/{2}+\epsilon ){n^{2}}/{2}$ edges such that the size of the largest bi-clique in $\overline{G}$ is $O((\log n)/{\epsilon })$, see [9].

Finally, the existence of linear sized bi-cliques in the complement of not too dense string graphs also follows from a recent graph theoretic result of Chudnovsky et al. [2]:

### Theorem 1.3

For every graph *H*, there exists $\epsilon >0$ such that if *G* is a graph with *n* vertices and at most $\epsilon n^{2}$ edges, and *G* does not contain a subdivision of *H* as an induced subgraph, then $\overline{G}$ contains a bi-clique of size at least $\epsilon n$.

Indeed, if *G* is a string graph, then *G* does not contain a subdivision of $K_{5}$ as an induced subgraph (we discuss this in more detail below). In order to prove Theorem 1.2, we shall also consider graphs which avoid certain weaker notions of subdivisions of $K_{5}$ as an induced subgraph.

Our paper is organized as follows. In Sect. 2, we prove Theorem 1.2. In Sect. 3, we conclude our paper with some open problems and remarks. But first, let us agree on some terminology.

### Preliminaries and Notation

For a positive integer *t*, $K_{t}$ denotes the complete graph on *t* vertices. Let *G* be a graph. If $v\in V(G)$, then $N(v)=\{w\in V(G):vw\in E(G)\}$ is the neighborhood of *v*. Also, if *U* is a subset of the vertices, then *G*[*U*] is the subgraph of *G* induced by *U*. Moreover, if *U* and *V* are disjoint subsets of *V*(*G*), then *E*(*U*, *V*) is the set of edges in *G* with one endpoint in *U* and one endpoint in *V*.

## Proof of Theorem 1.2

In this section we prove Theorem 1.2. First, we shall reduce Theorem 1.2 to a purely graph theoretic statement. In order to do this, we make use of the following immediate consequence of Theorem 1.1.

### Lemma 2.1

There exists a constant $C>0$ such that for every positive integer *n*, if *G* is a string graph with *n* vertices and at most $Cn^{2}$ edges, then $\overline{G}$ contains a bi-clique of size at least *n*/4.

Note that the constant 1/4 has no significance in this lemma, any positive constant would serve our purposes. Therefore, instead of citing Theorem 1.1, we could have cited Theorem 1.3 as well to get somewhat weaker version of this lemma. In the rest of the paper, *C* denotes the constant described by Lemma 2.1. This lemma tells us that in order to prove Theorem 1.2, it is enough to consider dense graphs. But for dense graphs, we can use powerful tools such as the regularity lemma.

Let us introduce some of the main notions used in this section. Let *G* be a graph with *n* vertices. If $0<\delta <{1}/{2}$, we say that *G* is $\delta$*-full* if for every $A,B\subset V(G)$ satisfying $A\cap B=\emptyset$ and $|A|,|B|\ge \delta n$, there exists an edge between *A* and *B*. The *density* of *G* is defined as $d(G)={|E(G)|}/{n^{2}}$ (which differs from the usual definition of $${|E(G)|}/{\left( {\begin{array}{c}n\\ 2\end{array}}\right) }$$). Say that *G* is $(\alpha ,\beta )$*-dense* if every induced subgraph of *G* with at least $\alpha n$ vertices has density at least $\beta$. We get the following immediate corollary of Lemma 2.1.

### Corollary 2.2

If *G* is a string graph that is $\delta$-full, then *G* is $(4\delta ,C)$-dense.

### Proof

Indeed, if *G* is not $(4\delta ,C)$-dense, then there exists $U\subset V(G)$ such that $|U|\ge 4\delta n$ and ${e(G[U])}/{|U|^{2}}=d(G[U])<C$. But then, by Lemma 2.1, $\overline{G}[U]$ contains a bi-clique of size at least ${|U|}/{4}\ge \delta n$, so *G* is not $\delta$-full. $\square$

If *H* is a graph, a *k-subdivision* of an edge $xy\in E(H)$ is the following operation: we replace the edge *xy* by a path $x=x_{0},x_{1},\dots ,x_{k+1}=y$, where $\{x_{1},\dots ,x_{k}\}$ is disjoint from *V*(*H*). A subdivision of an edge is a *k*-subdivision for some $k\ge 1$. A graph $H'$ is a *k*-subdivision (or subdivision) of *H* if we get $H'$ from *H* by *k*-subdividing (or subdividing) every edge of *H*. Also, $H'$ is a *partial subdivision* of *H* if we get $H'$ from *H* by subdividing some (possibly zero) edges of *H*. If $H'$ is a partial subdivision of *H*, we refer to the vertices of *H* in $H'$ as *branch-vertices*, and we refer to the other vertices as *side-vertices*.

**Fig. 2:**
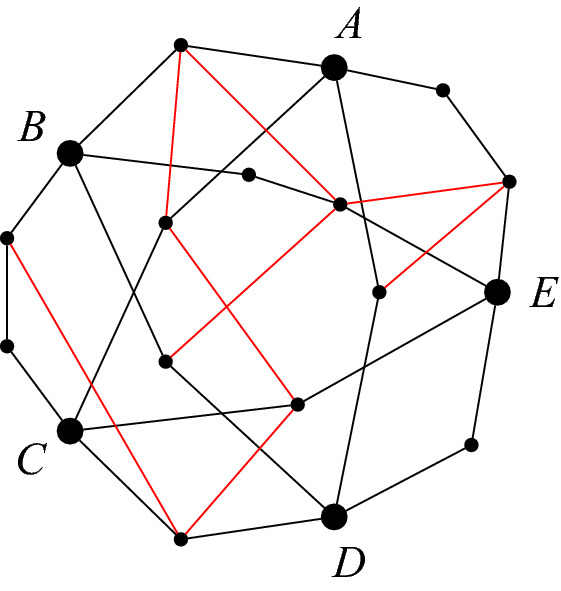
A weak subdivision of $K_{5}$, where *A*, *B*, *C*, *D*, *E* are the branch-vertices. The edges that are part of the weak subdivision but not the subdivision are red

If $H'$ is a *k*-subdivision (or subdivision) of *H*, let $H''$ be a graph we get from $H'$ by adding edges between some pairs of side-vertices that belong to the subdivisions of adjacent edges of *H*. Then $H''$ is called a *weak k-subdivision* (or weak subdivision) of *H*. More precisely, $H''$ is a *weak k-subdivision* (or weak subdivision) of *H* if there exists a *k*-subdivision (or subdivision) $H'$ of *H* such that ${V(H'')=V(H')}$, $E(H')\subset E(H'')$, and for every $uv\in E(H'')\setminus E(H')$, *u* and *v* are side-vertices of $H'$, and if *u* belongs to the subdivision of the edge $xy\in E(H)$ and *v* belongs to the subdivision of $x'y'\in E(H)$, then $xy\ne x'y'$ and $xy,x'y'$ are adjacent. See Fig. 2 for an example.

Slightly generalizing [17, Lem. 3.2] and [15, Lem. 11], we show that string graphs do not contain weak subdivisions of $K_{5}$.

### Lemma 2.3

If *G* is a string graph, then *G* does not contain a weak subdivision of $K_{5}$ as an induced subgraph.

### Proof

It is enough to show that weak subdivisions of $K_{5}$ are not string graphs. Suppose, to the contrary, that a string graph $H'$ is a weak subdivision of $K_{5}$. Let $\mathscr {C}$ be a collection of curves realizing $H'$. Let $v_{1},\dots ,v_{5}$ be the branch-vertices of $H'$ and let $C_{i}\in \mathscr {C}$ be the curve corresponding to $v_{i}$ for $i=1,\dots ,5$. Let $x_{i}$ be an arbitrary point of $C_{i}$. For every $1\le i<j\le 5$, there exists a path connecting $v_{i}$ and $v_{j}$ in $H'$. Let $X_{i,j}$ be the union of the curves in $\mathscr {C}$ corresponding to the vertices of this path. Then $X_{i,j}$ is a connected set in the plane containing $x_{i}$ and $x_{j}$, so there exists a curve $\gamma _{i,j}\subset X_{i,j}$ with endpoints $x_{i}$ and $x_{j}$. Note that as $H'$ is a weak subdivision, $X_{i,j}\cap X_{i',j'}\ne \emptyset$ if and only if $\{i,j\}\cap \{i',j'\}\ne \emptyset$. Hence, $\bigcup _{1\le i<j\le 5}\gamma _{i,j}$ is a drawing of $K_{5}$ in which if two edges cross, then they are adjacent. However, $K_{5}$ is not planar, so there exists no such drawing by an old theorem of Hanani (Chojnacki) [3] and Tutte [21]. $\square$

By combining Lemmas 2.1 and 2.3, in order to prove Theorem 1.2 it is enough to prove the following graph theoretic statement.

### Theorem 2.4

For every $\epsilon >0$ there exists $\delta >0$ such that the following holds. Let *G* be a graph with *n* vertices and at most $({1}/{4}-\epsilon ){n^{2}}/{2}$ edges such that *G* is $(4\delta ,C)$-dense and $\delta$-full. Then *G* contains a weak subdivision of $K_{5}$.

The rest of this section is devoted to a proof of Theorem 2.4. Let us briefly outline it. With the help of the regularity lemma, we partition the vertex set of *G* into a constant number of sets so that the bipartite graphs induced by most pairs of these sets are random-like. The notion of regularity and the regularity lemma are described in Sect. 2.1. Given a partial subdivision *H* of $K_{5}$, we show that an induced weak subdivision of *H* can be found in *G* if we can find |*V*(*H*)| parts in the partition of *G* satisfying certain properties. This argument is presented in Sect. 2.2. Then, we finish our proof in Sect. 2.3 by showing that a regular partition of *G* must contain |*V*(*H*)| parts with the desired properties.

### Regularity Lemma

In this section, we review the notion of regularity and state the regularity lemma. If *G* is a graph and $A,B\subset V(G)$, let

$$\begin{aligned} d(A,B)=\frac{|E(A,B)|}{|A|\cdot |B|}. \end{aligned}$$

For $\lambda >0$, a pair of subsets (*A*, *B*) of *V*(*G*) is $\lambda$*-regular*, if for every $A'\subset A$ and $B'\subset B$ satisfying $|A'|\ge \lambda |A|$ and $|B'|\ge \lambda |B|$, we have

$$|d(A,B)-d(A',B')|\le \lambda .$$

A $\lambda$-regular partition of the graph *G* is a partition $V(G)= V_{1}\cup \ldots \cup V_{k}$ such that

- $$|V_{1}|,\dots ,|V_{k}|\in \{\lfloor {n}/{k}\rfloor ,\lceil {n}/{k}\rceil \}$$,
- all but at most $\lambda k^{2}$ of the pairs $(V_{i},V_{j})$ are $\lambda$-regular.

#### Regularity Lemma

(Szemerédi [19]) For every $\lambda >0$ and positive integer *m* there exists a positive integer *M* such that the following holds. Let *G* be a graph, then *G* has a $\lambda$-regular partition into *k* parts, where $m\le k \le M$.

Given a graph *G* with $\lambda$-regular partition $(V_{1},\dots ,V_{k})$, the *reduced graph* of this partition is the edge-weighted graph (*R*, *w*), where $w:E(R)\rightarrow [0,1]$ is defined as follows.

- The vertex set of *R* is [*k*],
- *i* and *j* are joined by an edge if $(V_{i},V_{j})$ is $\lambda$-regular,
- if $$ij\in E(R)$$, then $$w(ij)=d(V_{i},V_{j})$$.

### Embedding

Let (*R*, *w*) be a complete edge-weighted graph and let $\epsilon _{1}>0$. An edge $xy\in E(R)$ is $\epsilon _{1}$*-thin* if $w(xy)\le \epsilon _{1}$, and *xy* is $\epsilon _{1}$*-fat* if $w(xy)\ge 1-\epsilon _{1}$. Let *H* be a graph with |*V*(*R*)| vertices. Say that (*R*, *w*) is $(H,\epsilon _{1})$*-admissible* if there exists a bijection $b:V(H)\rightarrow V(R)$ and a total ordering $\prec$ of the vertices of *H* such that

(i) no edge of *R* is $\epsilon _{1}$-fat,
(ii) if $xy\in E(H)$ is such that $x\prec y$ and $b(x)b(y)$ is $\epsilon _{1}$-thin, then $w(b(x)b(z))+w(b(y)b(z))<1-\epsilon _{1}$ holds for every $z\in V(H)\setminus \{x,y\}$ satisfying $x\prec z$.

Say that *b* and $\prec$ *witness* that (*R*, *w*) is $(H,\epsilon _{1})$-admissible if they satisfy the properties above. This section is devoted to the proof of the following embedding lemma.

#### Lemma 2.5

Let *H* be a graph with *h* vertices, let *k* be a positive integer, and let $\alpha ,\beta ,\delta ,\lambda ,\epsilon _{1}>0$ be real numbers satisfying

$$\begin{aligned} \epsilon _{1}<\beta ,\quad \lambda<\frac{\beta }{4h}\biggl (\frac{\epsilon _{1}}{2}\biggr )^{\!2h^{2}},\quad \delta<\frac{1}{k}\biggl (\frac{\epsilon _{1}}{2}\biggr )^{\!2h^{2}},\quad \text {and}\quad \alpha <\frac{1}{k}\biggl (\frac{\epsilon _{1}}{2}\biggr )^{\!2h}. \end{aligned}$$

Let *G* be an $(\alpha ,\beta )$-dense, $\delta$-full graph with a $\lambda$-regular partition $(V_{1},\dots ,V_{k})$ and corresponding reduced graph (*R*, *w*). If (*R*, *w*) contains an $(H,\epsilon _{1})$-admissible subgraph, then *G* contains a weak 2-subdivision of *H* as an induced subgraph.

#### Proof

Let $N=\lfloor {n}/{k}\rfloor$ and, for simplicity, assume that $|V_{1}|=\ldots =|V_{k}|=N$. Let the vertex set of *H* be $\{v_{1},\dots ,v_{h}\}$ and let $R'$ be an $(H,\epsilon _{1})$-admissible subgraph of *R*. Without loss of generality, suppose that $\{1,\dots ,h\}$ is the vertex set of $R'$. Also, the bijection *b* and ordering $\prec$ witnessing that $R'$ is $(H,\epsilon _{1})$-admissible are defined as follows: $b(v_{i})=i$ for $i=1,\dots ,h$ and $v_{1}\prec \dots \prec v_{h}$. Let $H'$ be the 2-subdivision of *H*, and for every edge $v_{i}v_{j}\in E(H)$, let $s_{i,j}$ and $s_{j,i}$ be the two side-vertices in $H'$ 2-subdividing $v_{i}v_{j}$, where $s_{i,j}$ is joined to $v_{i}$ and $s_{j,i}$ is joined to $v_{j}$. We embed the vertices of $H'$ in *G* so that

- for $i=1,\dots ,h$, the image of $v_{i}$ is some vertex $v_{i}'\in V_{i}$, and for $v_{i}v_{j}\in E(H)$, the image of $s_{i,j}$ is some vertex $s_{i,j}'\in V_{i}$,
- the image of each edge of $H'$ is an edge,
- the image of each non-edge of $H'$ is a non-edge, with the exception that $s'_{i,j}$ and $s'_{i,j'}$ might be adjacent if $v_{i}v_{j},v_{i}v_{j'}\in E(H)$.

**Fig. 3:**
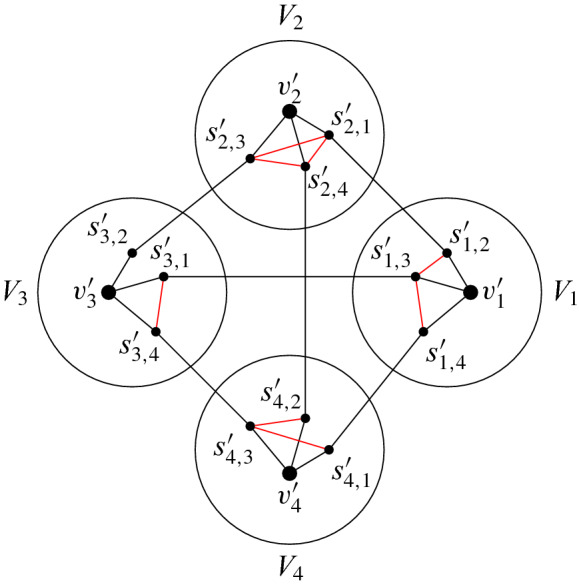
An embedding of a weak 2-subdivision of $K_{4}$. The edges that are part of the weak subdivision but not the subdivision are red

Clearly, such an embedding induces a weak 2-subdivision of *H* in *G*, so our task is reduced to showing that such an embedding exists. See Fig. 3 for an illustration.

Given a vertex $v\in V_{i}$ for some $i\in [h]$ and $h-1$ sets $U_{j}\subset V_{j}$ for $j\in [h]\setminus \{i\}$, say that *v* *is average with respect to* $(U_{j})_{j\in [h]\setminus \{i\}}$ if

$$\begin{aligned} \biggl |\frac{|N(v)\cap U_{j}|}{|U_{j}|}-w(ij)\biggr |<\lambda \end{aligned}$$

for $j\in [h]\setminus \{i\}$. The following useful claim is an easy consequence of the regularity condition.

#### Claim 2.6

Let $i\in [h]$, and for $j\in [h]\setminus \{i\}$, let $U_{j}\subset V_{j}$ be such that $|U_{j}|\ge \lambda N$. Then the number of vertices in $V_{i}$ that are not average with respect to $(U_{j})_{j\in [h]\setminus \{i\}}$ is at most $2h\lambda N$.

#### Proof

Let $W\subset V_{i}$ be the set of vertices not average with respect to $(U_{j})_{j\in [h]\setminus \{i\}}$. If $|W|\ge 2h\lambda N$, then there exist $j\in [h]\setminus \{i\}$ and $W'\subset W$ such that $|W'|\ge \lambda N$, and either ${|N(v)\cap U_{j}|}/{|U_{j}|}>w(ij)+\lambda$ for every $v\in W'$ or ${|N(v)\cap U_{j}|}/{|U_{j}|}<w(ij)-\lambda$ for every $v\in W'$. In the first case $d(W',U_{j})>d(V_{i},V_{j})+\lambda$, in the second case $d(W',U_{j})<d(V_{i},V_{j})-\lambda$, both contradicting the fact that $(V_{i},V_{j})$ is a $\lambda$-regular pair. $\square$

For $i=1,\dots ,h$, set $U_{i}:=V_{i}$. We embed the vertices of $H'$ in *G* step-by-step while updating the sets $U_{1},\dots ,U_{h}$ at the end of each step. During this procedure, say that an index $i\in [h]$ is *active*, if either $v_{i}'$ or $s_{i,j}'$ is not defined yet for some $v_{i}v_{j}\in E(H)$. During our procedure, the following properties are satisfied:

- at each step, we embed either one branch-vertex or two side-vertices connected by an edge,
- when a set is updated, it is replaced with one of its subsets,
- at the end of step *s*, $|U_{i}|\ge ({\epsilon _{1}}/{2})^{2s}N$ for $i\in [h]$,
- at the end of each step, if $v\in V(H')$ is already embedded in *G* and its image is $v'\in V_{i}$, then for every active index $j\in [h]\setminus \{i\}$ we have $N(v')\cap U_{j}=\emptyset$.

We analyze the first *h* steps and the rest of the steps separately.

*First* *h* *steps.* We embed the vertices $v_{1},\dots ,v_{h}$ one-by-one. More precisely, for $s=1,\dots ,h$, at step *s* we define the image $v'_{s}\in V_{s}$ of $v_{s}$ and update the sets $U_{1},\dots ,U_{h}$ so that, in addition to the properties above, the following condition is also satisfied:

- at the end of step *s*, we have $U_{s}\subset N(v'_{s})$.

We proceed at step *s* as follows. In the beginning of step *s*, we have $|U_{i}|\ge ({\epsilon _{1}}/{2})^{2s-2}N$ for $i\in [h]$. As *G* is $(\alpha ,\beta )$-dense and $|U_{s}|\ge ({\epsilon _{1}}/{2})^{2h}N\ge \alpha n$, we have $d(G[U_{s}])\ge \beta$. Let *W* be the set of vertices in $U_{s}$ whose degree in $G[U_{s}]$ is at least $\beta |U_{s}|$. Then

$$\begin{aligned} \beta |U_{s}|^{2}\le E(G[U_{s}])\le \frac{|W|\cdot |U_{s}|+\beta |U_{s}|^{2}}{2}, \end{aligned}$$

so $|W|\ge \beta |U_{s}|\ge \beta ({\epsilon _{1}}/{2})^{2h}N$. As $|W|> 2h\lambda N$ and $|U_{i}|\ge \lambda N$ for $i\in [h]$, we can apply Claim 2.6 to conclude that there exists $w\in W$ such that *w* is average with respect to $(U_{i})_{i\in [h]\setminus \{s\}}$. Note that for $i\in [h]\setminus \{s\}$, we have

$$\begin{aligned} |U_{i}\setminus N(w)|= & {} |U_{i}|-|U_{i}\cap N(w)|\ge |U_{i}|-|U_{i}|(w(si)+\lambda )\\\ge & {} |U_{i}|(\epsilon _{1}-\lambda )>\frac{\epsilon _{1}}{2}|U_{i}|> \biggl (\frac{\epsilon _{1}}{2}\biggr )^{\!2s}N, \end{aligned}$$

where the second inequality holds noting that *si* is not $\epsilon _{1}$-fat. Also,

$$\begin{aligned} |U_{s}\cap N(w)|\ge \beta |U_{s}|\ge \epsilon _{1}|U_{s}|>\biggl (\frac{\epsilon _{1}}{2}\biggr )^{\!2s}N. \end{aligned}$$

Set $v'_{s}=w$ and make the following updates: $U_{i}:=U_{i}\setminus N(w)$ for $i\in [h]\setminus \{s\}$ and $U_{s}:=N(w)\cap U_{s}$. This finishes step *s*.

*Rest of the steps.* Let $f_{1},\dots ,f_{l}$ be an enumeration of the edges of *H* such that if $1\le i<j\le l$ and $f_{i}=v_{a}v_{b}$, $f_{j}=v_{a'}v_{b'}$, where $a<b$, $a'<b'$, then $a\le a'$. Now for $s=1,\dots ,l$, we embed the two side-vertices on the 2-subdivision of the edge $f_{s}$ at step $h+s$.

We proceed at step $h+s$ as follows. In the beginning of the step, we have $|U_{i}|\ge ({\epsilon _{1}}/{2})^{2(h+s)-2}N$. Let $f_{s}=v_{a}v_{b}$, where $a<b$. Note that by the ordering of the edges, the active indices at this step are $i\in \{a,a+1,\dots ,h\}$. Consider two cases.

*Case 1:* $w(ab)\ge \epsilon _{1}$. As $|U_{a}|>({\epsilon _{1}}/{2})^{2h^{2}}N>2h\lambda N$ and $|U_{i}|\ge \lambda N$, we can apply Claim 2.6 to find a vertex $u\in U_{a}$ such that *u* is average with respect to $(U_{i})_{i\in [h]\setminus \{a\}}$. For $i\in [h]\setminus \{a\}$, let $U_{i}'=U_{i}\setminus N(u)$, and let $U_{a}'=U_{a}$. Then, by similar calculations as before, we have $|U_{i}'|\ge {\epsilon _{1}}|U_{i}|/2$. Also, let $W=U_{b}\cap N(u)$, then

$$|W|=|U_{b}\cap N(u)|\ge |U_{b}|(w(ab)-\lambda )\ge |U_{b}|(\epsilon _{1}-\lambda )\ge |U_{b}|\frac{\epsilon _{1}}{2}.$$

But then $|W|> 2h\lambda N$ and $|U'_{i}|\ge \lambda N$, so by Claim 2.6 there exists $w\in W$ such that *w* is average with respect to $(U'_{i})_{i\in [h]\setminus \{b\}}$. But then for $i\in [h]\setminus \{b\}$, by the same calculations as before, we have

$$|U_{i}'\setminus N(w)|\ge \frac{\epsilon _{1}}{2}|U_{i}'|\ge \biggl (\frac{\epsilon _{1}}{2}\biggr )^{\!2}|U_{i}|\ge \biggl (\frac{\epsilon _{1}}{2}\biggr )^{\!2(h+s)}N.$$

Set $s_{a,b}'=u$, $s_{b,a}'=w$, and make the following updates: $U_{i}:=U_{i}'\setminus N(w)$ for $i\in [h]\setminus \{b\}$ and $U_{b}:=U_{b}'$. This finishes step $h+s$.

*Case 2:* $w(ab)<\epsilon _{1}$. Let $W_{a}$ be the set of elements in $U_{a}$ that are average with respect to $(U_{i})_{i\in [h]\setminus \{a\}}$, and let $W_{b}$ be the set of elements in $U_{b}$ that are average with respect to $(U_{i})_{i\in [h]\setminus \{b\}}$. As $|U_{i}|\ge \lambda N$, we get by Claim 2.6 that $|W_{a}|\ge |U_{a}|-2h\lambda N\ge |U_{a}|/2$ and $|W_{b}|\ge |U_{b}|/2$. But then $|W_{a}|,|W_{b}|>({\epsilon _{1}}/{2})^{2h^{2}}N>\delta n$ and *G* is $\delta$-full, so there exists an edge between $W_{a}$ and $W_{b}$. Let $w_{a}\in W_{a}$ and $w_{b}\in W_{b}$ be the endpoints of such an edge. For every $i\in \{a+1,\dots ,h\}\setminus \{b\}$, let $U_{i}'=U_{i}\setminus (N(w_{a})\cup N(w_{b}))$. Then

$$\begin{aligned} |U_{i}'|= & {} |U_{i}|-|N(w_{a})\cup N(w_{b})|\ge |U_{i}|-|N(w_{a})|-|N(w_{b})|\\\ge & {} |U_{i}|-(w(ai)+\lambda )|U_{i}|-(w(bi)+\lambda )|U_{i}|. \end{aligned}$$

Note that we have $w(ai)+w(bi)\le 1-\epsilon _{1}$ as $R'$ is $(H,\epsilon _{1})$-admissible and $a<i$. Hence,

$$|U_{i}'|\ge |U_{i}|(\epsilon _{1}-2\lambda )\ge |U_{i}|\frac{\epsilon _{1}}{2}\ge \biggl (\frac{\epsilon _{1}}{2}\biggr )^{\!2(h+s)}N.$$

Set $s_{a,b}'=w_{a}$, $s_{b,a}'=w_{b}$, and make the following updates: $U_{i}:=U_{i}'$ if $i\in \{a+1,\dots ,h\}\setminus \{b\}$, $U_{a}:=U_{a}\setminus N(w_{b})$, $U_{b}:=U_{b}\setminus N(w_{a})$, and do not update the sets $U_{1},\dots ,U_{a-1}$. This finishes step $h+s$.

It is easy to check that at the end of the procedure we get the desired embedding of $H'$ in *G*. $\square$

### Finding an Admissible Subgraph

Given an edge-weighted graph (*R*, *w*), the *weight* of *R* is defined as $w(R)=\sum _{f\in E(R)}w(f)$. The aim of this section is to prove the following lemma.

#### Lemma 2.7

Let $\mathscr {H}$ be the family of partial subdivisions of $K_{5}$ with at most eight vertices. For every $\epsilon _{2}>0$ there exist $k_{0}=k_{0}(\epsilon _{2})\in \mathbb {N}^{+}$ and $\epsilon _{1}=\epsilon _{1}(\epsilon _{2})>0$ such that the following holds for every $k\ge k_{0}$. Let (*R*, *w*) be an edge-weighted graph with *k* vertices, at least ${k^{2}}(1-\epsilon _{1})/{2}$ edges, and such that $w(R)\le {k^{2}}({1}/{4}-\epsilon _{2})/{2}$. Then there exists $H\in \mathscr {H}$ such that *R* contains an $(H,\epsilon _{1})$-admissible subgraph.

Note that a weak subdivision of a partial subdivision of $K_{5}$ might not be a weak subdivision of $K_{5}$, but it contains a weak subdivision of $K_{5}$ as an induced subgraph. Therefore, it is enough to find an $(H,\epsilon _1)$-admissible subgraph of the reduced graph for some $H\in \mathscr {H}$ in order to be able to apply Lemma 2.5. Also, we limit the number of vertices of the members of $\mathscr {H}$ so we can view the parameter $h=|V(H)|$ as a constant.

Given an edge-weighted graph (*R*, *w*), let $R_{0}$ be the *complete* graph on *V*(*R*), and define the weight function $w_{0}:E(R_{0})\rightarrow [0,1]$ as follows. For every $f\in E(R_{0})$, let

$$\begin{aligned} w_{0}(f)={\left\{ \begin{array}{ll} \max {(w(f)+\epsilon _{1},1)} &{}\text{ if } f\in E(R) \text{ and } w(f)> \epsilon _{1},\\ 0 &{}\text{ if } f\in E(R) \text{ and } w(f)\le \epsilon _{1},\\ 1 &{}\text{ if } f\notin E(R). \end{array}\right. } \end{aligned}$$

This choice of weight function satisfies that for every graph *H*, an (*H*, 0)-admissible subgraph of $(R_{0},w_{0})$ is $(H,\epsilon _{1})$-admissible in (*R*, *w*). Also, if $(1-\epsilon _{1}){|V(R)|^{2}}/2 \le |E(R)|$, then $|w(R)-w_{0}(R_{0})|<2\epsilon _{1}|V(R)|^{2}$. Therefore, in order to prove Lemma 2.7, it is enough to prove the following lemma.

#### Lemma 2.8

Let $\mathscr {H}$ be the family of partial subdivisions of $K_{5}$ with at most eight vertices. For every $\epsilon _{3}>0$ there exists $k_{1}=k_{1}(\epsilon _{3})\in \mathbb {N}^{+}$ such that the following holds for every $k\ge k_{1}$. Let (*R*, *w*) be a complete edge-weighted graph with *k* vertices such that $w(R)\le {k^{2}}({1}/{4}-\epsilon _{3})/{2}$. Then there exists $H\in \mathscr {H}$ such that *R* contains an (*H*, 0)-admissible subgraph.

#### Proof of Lemma 2.7 assuming Lemma 2.8

Let $\epsilon _{3}={\epsilon _{2}}/{2}$ and let $k_{1}=k_{1}(\epsilon _{3})$ be the constant given by Lemma 2.8. We show that the choice $k_{0}=k_{1}$ and $\epsilon _{1}={\epsilon _{2}}/{8}$ suffices. Let (*R*, *w*) be an edge-weighted graph with $k\ge k_{0}$ vertices, at least ${k^{2}}(1-\epsilon _{1})/{2}$ edges such that $w(R)\le {k^{2}}({1}/{4}-\epsilon _{2})/{2}$, and define the complete edge-weighted graph $(R_{0},w_{0})$ as above. Then $w_{0}(R_{0})\le w(R)+2\epsilon _{1}k^{2}\le {k^{2}}({1}/{4}-\epsilon _{3})/{2}.$ Hence, $(R_{0},w_{0})$ contains an (*H*, 0)-admissible subgraph for some $H\in \mathscr {H}$. But then this subgraph is also $(H,\epsilon _{1})$-admissible in (*R*, *w*). $\square$

As a reminder, a complete subgraph $R'$ of the complete edge-weighted graph (*R*, *w*) is (*H*, 0)-admissible for some graph *H* if $|V(R')|=|V(H)|$, and there exist a bijection $b:V(H)\rightarrow V(R')$ and a total ordering $\prec$ of the vertices of *H* such that

(i) $R'$ contains no edge of weight 1,
(ii) if $xy\in E(H)$ is such that $x\prec y$ and $w(b(x)b(y))=0$, then $w(b(x)b(z))+w(b(y)b(z))<1$ holds for every $z\in V(H)\setminus \{x,y\}$ satisfying $x\prec z$.

In what comes, fix a positive integer *k* and a finite family of graphs $\mathscr {H}$. Let (*R*, *w*) be a *complete* edge-weighted graph on *k* vertices that does not contain an (*H*, 0)-admissible subgraph for any $H\in \mathscr {H}$. Also, among all such weighted graphs, choose those that minimize the weight *w*(*R*), and among those, choose one that minimizes the size of the set $F_{w}=\{w(f):f\in E(R)\}\setminus \{0,{1}/{2},1\}$. Note that the set of weight functions $w:E(R)\rightarrow [0,1]$ for which (*R*, *w*) does not contain an (*H*, 0)-admissible subgraph for any $H\in \mathscr {H}$ is a compact subset of $\mathbb {R}^{E(R)}$, so *w*(*R*) attains its minimum on this set. We show that (*R*, *w*) must have a simple structure, reminiscent of the extremal graphs given by the classical Turán’s theorem [20], not containing a clique of given size. To show this, we proceed by following a similar train of thoughts as one of the traditional proofs of Turán’s theorem.

#### Proposition 2.9

$$F_{w}=\emptyset $$.

#### Proof

Say that a triple of vertices (*x*, *y*, *z*) in (*R*, *w*) is *dangerous* if $w(xy)=0$ and $w(xz)+w(yz)\ge 1$, and let $D_{w}$ be the set of dangerous triples. Also, let $A_{w}=\{e\in E(R):w(e)=1\}$. We show that if $F_{w}$ is non-empty, then this contradicts the minimality of *w*, or the minimality of $F_{w}$. More precisely, we show that we can replace the weight function *w* with $w'$ such that $w'(R)\le w(R)$, $|F_{w'}|<|F_{w}|$, $D_{w}\subset D_{w'}$, and $A_{w}\subset A_{w'}$. First, we show that the latter two conditions imply that if (*R*, *w*) has no (*H*, 0)-admissible subgraph, then $(R,w')$ has no (*H*, 0)-admissible subgraph either.

Indeed, suppose that *Q* is a subgraph of *R* such that $(Q,w')$ is an (*H*, 0)-admissible subgraph with *b* and $\prec$ witnessing, then we show that $(Q,w')$ is (*H*, 0)-admissible with *b* and $\prec$ witnessing. By (i), $(Q,w')$ has no edge of weight 1, but as $E_{w}\subset E_{w'}$, no edge of (*Q*, *w*) has weight 1 either. Therefore, (i) holds in (*Q*, *w*) as well. Suppose that (ii) does not hold in (*Q*, *w*), then there exist vertices $x,y,z\in V(H)$ such that $xy\in E(H)$, $x\prec y$, $x\prec z$, $w(b(x)b(y))=0$, and $w(b(x)b(z))+w(b(y)b(z))\ge 1$. But then $(b(x),b(y),b(z))\in D_{w}$, so $(b(x),b(y),b(z))\in D_{w'}$ as well, which means that $w'(b(x)b(z))+w'(b(y)b(z))\ge 1$. This contradicts the fact that $(Q,w')$ is (*H*, 0)-admissible with *b* and $\prec$ witnessing.

Now let us show how to construct $w'$ satisfying the properties above. For $r\in [0,1]$, let $F(r)=\{f\in E(R):w(f)=r\}$. Suppose that $F_{w}$ is non-empty and let $r\in F_{w}$. Distinguish between two cases.

*Case 1:* $1-r\notin F_{w}$. If $r<{1}/{2}$, let *q* be the largest real number smaller than *r* such that $q\in F_{w}$ or $1-q\in F_{w}$; if there exists no such *q*, let $q=0$. If $r>{1}/{2}$, let *q* be the largest real number smaller than *r* but not less than 1/2 such that $q\in F_{w}$ or $1-q\in F_{w}$; if there exists no such *q*, let $q={1}/{2}$. Define the new weight function $w'$ as

$$w'(f)={\left\{ \begin{array}{ll}q &{}\text{ if } f\in F(r),\\ w(f)&{}\text{ if } f\in E(R)\setminus F(r).\end{array}\right. }$$

Then clearly none of the weights increase, but some of the weights strictly decrease, so $w'(R)<w(R)$. Also, $F_{w'}=F_{w}\setminus \{r\}$ and $A_{w'}=A_{w}$. It remains to show that $D_{w}\subset D_{w'}$ also holds. Let $(x,y,z)\in D_{w}$, then $0=w(xy)=w'(xy)$ and $w(xz)+w(yz)\ge 1$. If $w(xz)=w'(xz)$ and $w(yz)=w'(yz)$, then clearly $(x,y,z)\in D_{w'}$. Therefore, without loss of generality, suppose that $w(xz)\ne w'(xz)$, the case $w(yz)\ne w'(yz)$ can be handled similarly. Then $w(xz)=r$ and $w'(xz)=q$. Also, $w(yz)\ge 1-r$, but the smallest number in $F_{w}$ which is at least $1-r$ is at least $1-q$, so $w(yz)\ge 1-q$. If $w(yz)=w'(yz)$, then this implies $(x,y,z)\in D_{w'}$. If $w(yz)\ne w'(yz)$, then $w(yz)=r>{1}/{2}$, and $w'(xz)=w'(yz)=q\ge {1}/{2}$, so $(x,y,z)\in D_{w'}$ as well.

*Case 2:* $1-r\in F_{w}$. Without loss of generality, we can suppose that $|F(r)|\ge |F(1-r)|$. Again, if $r<{1}/2$, let *q* be the largest real number smaller than *r* such that $q\in F_{w}$ or $1-q\in F_{w}$; if there exists no such *q*, let $q=0$. If $r>1/{2}$, let *q* be the largest real number smaller than *r* but not less than 1/2 such that $q\in F_{w}$ or $1-q\in F_{w}$; if there exists no such *q*, let $q={1}/{2}$. Define the new weight function $w'$ as

$$w'(f)={\left\{ \begin{array}{ll}q &{}\text{ if } f\in F(r),\\ 1-q &{}\text{ if } f\in F(1-r),\\ w(f)&{}\text{ if } f\in E(R)\setminus (F(r)\cup F(1-r)).\end{array}\right. }$$

Then $w'(R)=w(R)+(q-r)(|F(r)|-|F(1-r)|)\le w(R)$, $F_{w'}=F_{w}\setminus \{r,1-r\}$, and $A_{w}\subset A_{w'}$. Again, we show that $D_{w}\subset D_{w'}$ holds as well. Let $(x,y,z)\in D_{w}$, then $0=w(xy)=w'(xy)$. If $w(xz)=w'(xz)$ and $w(yz)=w'(yz)$, then clearly $(x,y,z)\in D_{w'}$. Therefore, without loss of generality, suppose that $w(xz)\ne w'(xz)$; the case $w(yz)\ne w'(yz)$ can be handled similarly. Then $w(xz)\in \{r,1-r\}$. If $w(xz)+w(yz)=1$, then either $w(xz)=r$ and $w(yz)=1-r$, or $w(xz)=1-r$ and $w(yz)=r$. In the first case $w'(xz)=q$ and $w'(yz)=1-q$, in the second case $w'(xz)=1-q$ and $w'(yz)=q$. In both cases $w'(xz)+w'(yz)=1$, so $(x,y,z)\in D_{w'}$. On the other hand, consider the case $w(xz)+w(yz)>1$. If $w(xz)=r$, then $w(yz)>1-r$, but the smallest number larger than $1-r$ in $F_w$ is at least $1-q$, so $w(yz)\ge 1-q$. If $w'(yz)=w(yz)$, then it follows that $(x,y,z)\in D_{w'}$. If $w'(yz)\ne w(yz)$, then $w(yz)=r>{1}/{2}$ and $w'(yz)=w'(xz)=q\ge {1}/{2}$, so $(x,y,z)\in D_{w'}$ as well. The remaining case when $w(xz)=1-r$ can be handled in a similar manner. $\square$

#### Proposition 2.10

Among the complete edge-weighted graphs (*R*, *w*) on *k* vertices, where $w:E(R)\rightarrow \{0,1/2,1\}$ and (*R*, *w*) has no (*H*, 0)-admissible subgraph for any $H\in \mathscr {H}$, there is one with minimum weight *w*(*R*) satisfying the following properties. The vertex set of *R* can be partitioned into parts $X_1,\dots ,X_s$, for some $s\in \mathbb N$, such that for all $i\in [s]$ every edge in $X_{i}$ (possibly no edges) has weight 1, and for every $1\le i<j\le s$, either every edge between $X_{i}$ and $X_{j}$ has weight 1/2, or every edge between $X_{i}$ and $X_{j}$ has weight 0.

#### Proof

Let (*R*, *w*) be a complete edge-weighted graph satisfying conditions desired in the proposition. For $x\in V(R)$, let $d_{w}(x)=\sum _{y\in V(R)\setminus \{x\}}w(xy)$. For $x,y\in V(R)$, let $x\sim y$ if $w(xy)=1$. We show that $\sim$ is an equivalence relation. Suppose not, then there exist $x,y,z\in V(R)$ such that $x\sim y$, $y\sim z$, but $x\not \sim z$. Consider two cases.

*Case 1:* $d_{w}(y)>d_{w}(x)$ or $d_{w}(y)>d_{w}(z)$. Assume that $d_{w}(y)>d_{w}(x)$, the other case being similar. Define a new weight function

$$w'(f)={\left\{ \begin{array}{ll}w(xu) &{}\text{ if } f=yu \text{ for } \text{ some } u\in V(R),\\ w(f) &{}\text{ otherwise. }\end{array}\right. }$$

Then $w'(R)=w(R)+d_{w}(x)-d_{w}(y)<w(R)$ and $(R,w')$ does not contain an (*H*, 0)-admissible subgraph for any $H\in \mathscr {H}$. Indeed, (*R*, *w*) and $(R,w')$ differ only in the edges containing *y*. Hence, if $(R,w')$ contains an (*H*, 0)-admissible subgraph $R'$, then $y\in V(R')$. But as $w'(xy)=1$, we must have $x\notin V(R')$. The weight distributions on the neighborhoods of *x* and *y* are identical in $(R,w')$, so then $R'\setminus \{y\}\cup \{x\}$ is also (*H*, 0)-admissible in both (*R*, *w*) and $(R,w')$, a contradiction.

*Case 2:* $d_{w}(y)\le d_{w}(x)$ and $d_{w}(y)\le d_{w}(z)$. Define the new weight function

$$w'(f)={\left\{ \begin{array}{ll}w(yu) &{} \text{ if } f=xu \text{ or } f=zu \quad \text{ for } \text{ some } u\in V(R),\\ w(f) &{}\,\text{ otherwise. }\end{array}\right. }$$

Then $w'(R)=w(R)+(2d_{w}(y)-1)-(d_{w}(x)+d_{w}(z)-w(xz))<w(R)$. Also, by a similar argument as in the previous case, $(R,w')$ does not contain an (*H*, 0)-admissible subgraph for any $H\in \mathscr {H}$. Indeed, (*R*, *w*) and $(R,w')$ differ only in the edges containing *x* or *u*. Hence, if $(R,w')$ contains an (*H*, 0)-admissible subgraph $R'$, then $x\in V(R')$ or $z\in V(R')$. As $w'(xu)=w'(xy)=1$, $V(R')$ cannot contain both *x* and *z*. Assume that $x\in V(R')$ and $z\notin V(R')$, the other case can be handled similarly. As $w'(xy)=1$, we must have $y\notin V(R')$. The weight distributions on the neighborhoods of *x* and *y* are identical in $(R,w')$, so then $R'\setminus \{x\}\cup \{y\}$ is also (*H*, 0)-admissible in both (*R*, *w*) and $(R,w')$, a contradiction.

Both cases contradict the minimality of *w*(*R*), so $\sim$ is an equivalence relation. Let $X_{1},\dots ,X_{s}$ be the equivalence classes of $\sim$. Now consider the following operation on weight functions. Let $w_{0}:E(R)\rightarrow \mathbb {R}^{+}$ be a weight function such that $w_{0}(xy)=1$ for every $x,y\in X_{i}$, $i=1,\dots ,s$. Define the weight function $C_{i}w_{0}$ on *E*(*R*) as follows. Let $x\in X_{i}$ be a vertex such that $d_{w_{0}}(x)$ is minimal among the vertices in $X_{i}$. Then for $f\in E(R)$, let

$$\begin{aligned} C_{i}w_{0}(f)={\left\{ \begin{array}{ll}w_{0}(xu) &{}\text{ if } f=yu \text{ for } \text{ some } y\in X_{i}, u\in V(R),\\ w_{0}(f) &{}\text{ otherwise. }\end{array}\right. } \end{aligned}$$

Then $C_{i}w_{0}(R)=w_{0}(R)+|X_{i}|d_{w_{0}}(x)-\sum _{y\in X_{i}}d_{w_{0}}(y)\le w_{0}(R)$. Also, for every graph *H*, if $(R,w_{0})$ does not have an (*H*, 0)-admissible subgraph, then $(R,C_{i}w_{0})$ also does not contain an (*H*, 0)-admissible subgraph. This holds by the same argument as in Case 1 above.

Let $w'=C_{s}C_{s-1}\dots C_{1}w$. Then $w'(R)\le w(R)$ and $(R,w')$ does not contain an (*H*, 0)-admissible subgraph for any $H\in \mathscr {H}$. Moreover, for any $1\le i<j\le s$, either every edge between $X_{i}$ and $X_{j}$ has weight 0, or every edge between $X_{i}$ and $X_{j}$ has weight 1/2. Indeed, it is easy to prove by induction on *l* that if $w_{l}=C_{l}C_{l-1}\dots C_{1}w$, then for every pair (*i*, *u*), where $1\le i\le l$ and $u\in X_{j}$ for some $j\in [s]\setminus \{i\}$, either for every $v\in X_{i}$ we have $w_{l}(uv)=0$, or for every $v\in X_{i}$ we have $w_{l}(uv)={1}/{2}$. But then $w'=w_{s}$ has the desired properties. $\square$

In what follows, suppose that (*R*, *w*) has the form described in Proposition 2.10. Define the *vertex*-weighted graph $(Q,\phi )$, where $\phi :V(Q)\rightarrow [0,1]$, as follows. Let $X_{1},\dots ,X_{s}$ be the partition of *V*(*R*) given by Proposition 2.10 and, as a reminder, $k=\sum _{i=1}^{s}|X_{i}|$. Then the vertex set of *Q* is [*s*], the weight of $a\in [s]$ is $\phi (a)={|X_{a}|}/{k}$, and *ab* is an edge of *Q* if every edge of *R* between $X_{a}$ and $X_{b}$ has weight 1/2. Note that $\phi$ is a probability distribution on [*s*], that is,

$$\begin{aligned} \sum _{a\in [s]}\phi (a)=1. \end{aligned}$$

We define the *weight* of the graph *Q* in the following unconventional way:

$$\begin{aligned} \phi (Q)=\sum _{a\in [s]} \phi (a)^{2}+\sum _{ab\in E(Q)}\!\!\phi (a)\phi (b). \end{aligned}$$

Note that with this definition, we have

1 $$\begin{aligned} w(R)=\sum _{xy\in E(R)}\!\!w(xy)=\sum _{i=1}^{s}\left( {\begin{array}{c}|X_{i}|\\ 2\end{array}}\right) +\sum _{ab\in E(Q)}\!\!\frac{|X_{a}|\cdot |X_{b}|}{2}=\frac{k^{2}}{2}\biggl (\phi (Q)-\frac{1}{k}\biggr ). \end{aligned}$$

Let *H* be a graph and let $Q'$ be an induced subgraph of *Q* on |*V*(*H*)| vertices. Say that $Q'$ is *H*-*admissible* if there exist a bijection $b:V(H)\rightarrow V(Q')$ and a total ordering $\prec$ of the vertices of *H* such that

- if $xy\in E(H)$ is such that $x\prec y$ and $b(x)b(y)\notin E(Q')$, then for every $z\in V(H)\setminus \{x,y\}$ satisfying $x\prec z$, either $b(x)b(z)\notin E(Q')$ or $b(y)b(z)\notin E(Q')$

Say that *b* and $\prec$ *witness* that $Q'$ is *H*-admissible if they satisfy the properties above. Note that *Q* contains an *H*-admissible subgraph if and only if (*R*, *w*) contains an (*H*, 0)-admissible subgraph. Hence, *Q* does not contain an *H*-admissible subgraph for any $H\in \mathscr {H}$.

First, we observe the following forbidden subgraph characterization of $K_{t}$-admissible graphs. The author is indebted to the referees for the next claim.

#### Claim 2.11

For every positive integer *t*, a *t* vertex graph *Q* is $K_{t}$-admissible if and only if *Q* does not contain a 4-cycle or a path of length 3 as an induced subgraph.

#### Proof

Note that *Q* is $K_{t}$-admissible if and only if there exists an ordering $\prec$ on *Q* such that for any three vertices *x*, *y*, *z*, if $xy,yz\in E(Q)$ and $xz\notin E(Q)$, then $y\prec x$ and $y\prec z$. It is easy to check that if *Q* contains an induced 4-cycle or an induced path of length 3, then such a labeling is not possible.

It remains to show that if *Q* does not contain an induced 4-cycle or an induced path of length 3, then we can find a suitable ordering $\prec$. We prove this by induction on *t*. If $t=1$, the statement is trivial, so assume that $t>1$. As *Q* does not contain an induced path of length 3, *Q* is a cograph,^2^ so we can partition *V*(*Q*) into two non-empty sets *A* and *B* such that either

- there are no edges between *A* and *B*, or
- every vertex in *A* is adjacent to every vertex in *B*.

Let $\prec _{A}$ and $\prec _{B}$ be suitable orderings of *Q*[*A*] and *Q*[*B*], respectively, which exist by our induction hypothesis. In the first case, let $\prec$ be any ordering whose restriction to *A* and *B* is $\prec _{A}$ and $\prec _{B}$, respectively. Then $\prec$ shows that *Q* is $K_{t}$-admissible.

Now consider the second case. If both *Q*[*A*] and *Q*[*B*] contain a non-edge, then *Q* contains an induced 4-cycle. Hence, *Q*[*A*] or *Q*[*B*] is a complete graph. Without loss of generality, suppose that *Q*[*A*] is complete. Then let $\prec$ be any ordering such that $a\prec b$ for every $a\in A$ and $b\in B$, and $\prec$ equals to $\prec _{B}$ on *B*. Then it is easy to check that $\prec$ is suitable. $\square$

The following proposition is the finishing touch in the proof of Lemma 2.8.

#### Proposition 2.12

Let $\mathscr {H}$ be the family of partial subdivisions of $K_{5}$ with at most eight vertices. If $(Q,\phi )$ has no *H*-admissible subgraph for any $H\in \mathscr {H}$, then $\phi (Q)\ge {1}/{4}.$

#### Proof

We prove this proposition through a series of claims. Assume that *Q* has no *H*-admissible subgraph for any $H\in \mathscr {H}$. First, we show that *Q* has at most seven vertices. We prepare the proof of this with the following claim.

#### Claim 2.13

If $s\ge 7$, the complement of *Q* has maximum degree 4.

#### Proof

Assume that there exist $a\in V(Q)$ and $B\subset V(Q)\setminus \{a\}$ such that $|B|=5$ and there are no edges between *a* and *B*. Let $b\in V(Q)\setminus (B\cup \{a\})$. Then there exists $A\subset B$ such that $|A|=3$ and either *b* is joined to every element of *A*, or there are no edges between *b* and *A*. In either case, $C=\{a,b\}\cup A$ does not contain a 4-cycle or a path of length 3 as an induced subgraph, so *Q*[*C*] is $K_{5}$-admissible by Claim 2.11. $\square$

#### Claim 2.14

$$s<8$$.

#### Proof

We show that if $s=8$, then *Q* contains an *H*-admissible subgraph for some $H\in \mathscr {H}$, then the same follows for all $s>8$ as well. By Observation 2.13, the minimum degree of *Q* is at least 3. First, suppose that there exists $v\in V(Q)$ with $\deg v=3$, and let *A* be the set of four vertices not adjacent to *v*. Then by Claim 2.11, *Q*[*A*] is either a path of length 3, or a cycle of length 4. In both cases, there exist three distinct vertices $a,b,c\in A$ such that $ab\notin E(Q)$ and $ac,bc\in E(Q)$. Let $B=(V(Q)\setminus (A\cup \{v\}))\cup \{a,b\}$. Then $|B|=5$, and as the minimum degree of *Q* is 3, both *a* and *b* have degree at least 1 in *Q*[*B*].

**Fig. 4:**
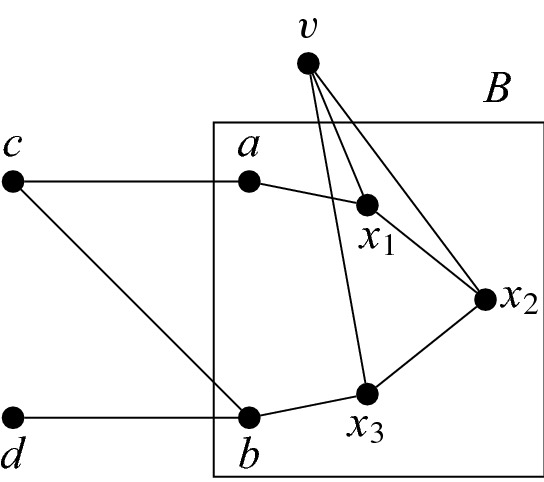
An illustration for the proof of Claim 2.14

We claim that there exists a path between *a* and *b* in *Q*[*B*]. Indeed, suppose not and let *C* be the connected component of *Q*[*B*] containing *a*. Then $|C|\ge 2$ and $|B\setminus C|\ge 2$ as both *a* and *b* have degree at least 1. But then $\{|C|,|B\setminus C|\}=\{2,3\}$, and there are no edges between *B* and $C\setminus B$. But then *Q*[*B*] does not contain a path of length 3 or a 4-cycle, which contradicts Claim 2.11 (Fig. 4).

Let $a=x_{0},x_{1},\dots ,x_{p+1}=b$ be the vertices of a path connecting *a* and *b* in *B* for some positive integer *p*, and set $D=A\cup \{v,x_{1},\dots ,x_{p}\}$. Also, let $K_{5}^{(p)}$ be the partial subdivision of $K_{5}$ in which one edge is *p*-subdivided. We show that *D* is $K_{5}^{(p)}$-admissible. Let 1, 2, 3, 4, 5 be the branch-vertices of $K_{5}^{(p)}$ and let $r_{1},\dots ,r_{p}$ be the vertices *p*-subdividing the edge 23. Let *c*, *d* be the two vertices in $A\setminus \{a,b\}$. Consider the vertex ordering on $K_{5}^{(p)}$ defined as $r_{1}\prec \cdots \prec r_{p}\prec 1\prec 2\prec 3\prec 4\prec 5$, and define the bijection *b* as $b(r_{i})=x_{i}$ for $i=1,\dots ,p$, $b(1)=v$, $b(2)=a$, $b(3)=b$, $b(4)=c$, $b(5)=d$. Then $\prec$ and *b* witness that *Q*[*D*] is $K_{5}^{(p)}$-admissible.

Now we can suppose that every vertex of *Q* has degree at least 4. Let $v\in V(Q)$ be an arbitrary vertex and let $A\subset N(v)$ be such that $|A|=4$. Again, by Claim 2.11, we have that *Q*[*A*] is either a cycle of length 4 or a path of length 3. Let $B=(V(Q)\setminus (A\cup \{v\}))\cup \{a,b\}$. Then $|B|=5$ and, as the minimum degree of *Q* is 4, both *a* and *b* have degree at least 1 in *Q*[*B*]. But then we can repeat the exact same argument as before to find a $K_{5}^{(p)}$-admissible subgraph for some $p\le 3$. This finishes the proof of the claim. $\square$

Therefore, in order to finish the proof of Proposition 2.12, it is enough to consider the cases $s=1,\dots ,7$. The following simple inequality will help us to deal with these few cases.

#### Claim 2.15

Let $$0\le a\le b\le c\le d$$. Then

$$\begin{aligned} ad+bc\le ac+bd\le ab+cd. \end{aligned}$$

#### Proof

The first inequality is equivalent to $(b-a)(d-c)\ge 0$, while the second inequality is equivalent to $(d-a)(c-b)\ge 0$. $\square$

Without loss of generality, suppose that $\phi (1)\le \phi (2)\le \ldots \le \phi (s)$. Now let us consider all possible values of *s*. For every value of *s*, we show that $\phi (Q)$ can be bounded from below by the sum of the squares of four numbers whose sum is equal to 1. But then $\phi (Q)\ge 1/4$.

$s\le 4$. In this case, we simply have

$$\phi (Q)\ge \sum _{i=1}^{s}\phi (i)^{2}\ge \frac{1}{s}\ge \frac{1}{4},$$

where the second inequality holds by the Cauchy–Schwarz inequality.

$s=5$. If *Q* is not $K_{5}$-admissible, then *Q* has more than two edges by Claim 2.11. But then

$$\begin{aligned} \phi (Q)\ge & {} \sum _{i=1}^{5}\phi (i)^{2}+2\phi (1)\phi (2)\\= & {} (\phi (1)+\phi (2))^{2}+\phi (3)^{2}+\phi (4)^{2}+\phi (5)^{2}\ge \frac{1}{4}, \end{aligned}$$

where the last inequality holds by the Cauchy–Schwarz inequality.

${s=6}$. By Claim 2.11, there exist three vertices $a,b,c\in \{2,3,4,5,6\}$ such that $ab,ac\in E(Q)$. But then $\phi (a)\phi (b)+\phi (a)\phi (c)\ge \phi (2)\phi (3)+\phi (2)\phi (4)$. Also, there are two disjoint edges $a_{1}b_{1},a_{2}b_{2}$ in $Q[[6]\setminus \{a\}]$. Then, by Claim 2.15, we have $\phi (a_{1})\phi (b_{1})+\phi (a_{2})\phi (b_{2})\ge \phi (1)\phi (4)+\phi (2)\phi (3)$. As $ab,ac,a_{1}b_{1},a_{2}b_{2}$ are four distinct edges of *Q*, we can write

$$\begin{aligned} \phi (Q)&\ge \sum _{i=1}^{6}\phi (i)^{2}+\phi (2)\phi (3)+\phi (2)\phi (4)+\phi (1)\phi (4)+\phi (2)\phi (3)\\&\ge (\phi (1)+\phi (4))^{2}+(\phi (2)+\phi (3))^{2}+\phi (5)^{2}+\phi (6)^{2}\ge \frac{1}{4}. \end{aligned}$$

${s=7}$. First, we show that if *Q* contains a cycle of length 6 or 7, we have $\phi (Q)\ge {1}/{4}$. Indeed, let $a_{1}<\ldots <a_{r}$ be the vertices of this cycle, where $r\in \{6,7\}$, and let $\pi \in S_{r}$ be a permutation such that $a_{\pi (1)}a_{\pi (2)},a_{\pi (2)}a_{\pi (3)},\dots , a_{\pi (r)}a_{\pi (1)}$ are the edges of this cycle. Then

$$\begin{aligned} \sum _{i=1}^{r}\phi (a_{\pi (i)})\phi (a_{\pi (i+1)})\ge & {} \sum _{i=1}^{r}\phi (a_{i})\phi (a_{r+1-i})\\\ge & {} 2\phi (1)\phi (6)+2\phi (2)\phi (5)+2\phi (3)\phi (4), \end{aligned}$$

where the first inequality is the consequence of the rearrangement inequality [11, Sect. 10.2, Thm. 368]. Hence,

$$\begin{aligned} \phi (Q)&\ge \sum _{i=1}^{7}\phi (i)^{2}+2\phi (1)\phi (6)+2\phi (2)\phi (5)+2\phi (3)\phi (4)\\&\ge (\phi (1)+\phi (6))^{2}+(\phi (2)+\phi (5))^{2}+(\phi (3)+\phi (4))^{2}+\phi (7)^{2}\ge \frac{1}{4}. \end{aligned}$$

By Claim 2.13, every vertex of *Q* has degree at least 2. Suppose that *Q* has a vertex *a* of degree 2, let $b_{1},b_{2}$ be the vertices adjacent to *a*, and let $C=\{c_{1},c_{2},c_{3},c_{4}\}$ be the set of all other vertices. By Claim 2.11, *Q*[*C*] is either a cycle of length 4 or a path of length 3.

*Case 1.* *C* is a cycle of length 4. Without loss of generality, let $\phi (c_{1})\le \phi (c_{2})\le \phi (c_{3})\le \phi (c_{4})$ and $\phi (b_{1})\le \phi (b_{2})$. Then

$$\begin{aligned} \phi (Q)&=\sum _{i=1}^{7}\phi (i)^{2}\,+\!\sum _{xy\in E(Q)}\!\!\phi (x)\phi (y)\\&\ge \sum _{i=1}^{7}\phi (i)^{2}+2\phi (c_{1})\phi (c_{4})+2\phi (c_{2})\phi (c_{3})+2\phi (a)\phi (b_{1})\\&=(\phi (c_{1})+\phi (c_{4}))^{2}+(\phi (c_{2})+\phi (c_{3}))^{2}+(\phi (a)+\phi (b_{1}))^{2}+\phi (b_{2})^{2}\ge \frac{1}{4}, \end{aligned}$$

where the first inequality holds by double application of Claim 2.15.

**Fig. 5:**
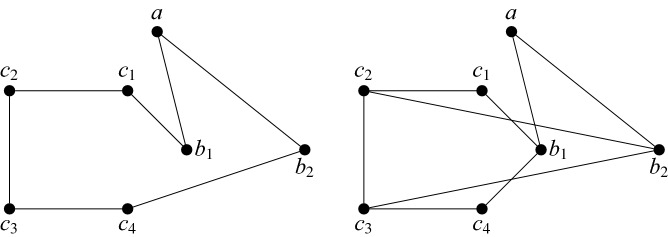
An illustration for Case 2. Left is the subcase where $c_{4}b_{2}$ is an edge, right is the subcase where $c_{4}b_{2}$ is not an edge

*Case 2.* *C* is a path of length 3. Without loss of generality, let the edges of this path be $c_{1}c_{2},c_{2}c_{3},c_{3}c_{4}$. As $c_{1}$ and $c_{4}$ both have degree at least 2, there is an edge from both $c_{1}$ and $c_{4}$ to $\{b_{1},b_{2}\}$. Without loss of generality, suppose that $c_{1}b_{1}$ is an edge. If $c_{4}b_{2}$ is an edge, then *Q* contains a cycle of length 7, namely $c_{1}c_{2}c_{3}c_{4}b_{2}ab_{1}$, so we are done. Hence, we may assume that $c_{4}b_{1}$ is an edge, and $c_{1}b_{2}$ and $c_{4}b_{2}$ are non-edges. But then both $b_{2}c_{2}$ and $b_{2}c_{3}$ are edges, because if $b_{2}c_{2}$ is a non-edge, say, then there are no edges between $\{a,b_{2}\}$ and $\{c_{1},c_{2},c_{4}\}$. Then $Q[\{a,b_2,c_1,c_2,c_4\}]$ is $K_{5}$-admissible by Claim 2.11, as it cannot contain a 4-cycle or path of length 3. But then *Q* contains a cycle of length 6, namely $c_{1}b_{1}c_{4}c_{3}b_{2}c_{2}$, so we are done. See Fig. 5.

Therefore, we have $\phi (Q)\ge {1}/{4}$ if *Q* contains a vertex with degree 2. Hence, we can suppose that every vertex has degree at least 3 in *Q*. As 7 is odd, *Q* has at least one vertex *a* of even degree, so the degree of *a* is either 4 or 6.

First, suppose that *a* has degree 4, let $B=\{b_{1},b_{2},b_{3},b_{4}\}$ be the neighbors of *a*, and $c_{1},c_{2}$ be the rest of the vertices. By Claim 2.11, *Q*[*B*] is either a cycle of length 4, or a path of length 3. In both cases $Q[B\cup \{a\}]$ contains a cycle *C* of length 5. If $c_{1}c_{2}$ is not an edge, then there are at least three edges between $c_{1}$ and *C*, so there are two consecutive vertices of *C* joined to $c_{1}$. But then $C\cup \{c_{1}\}$ contains a cycle of length 6, so we are done. Therefore, we can suppose $c_{1}c_{2}$ is an edge. Then there are at least two edges between $c_{i}$ and *C* for $i=1,2$. In this case, we can find two disjoint edges between $\{c_{1},c_{2}\}$ and *C*, which also implies the existence of a cycle of length at least 6 in *C*.

The only remaining case is when the degree of *a* is 6. Let $b\in V(Q)\setminus \{a\}$, and let *A* be the neighborhood of *b* in $V(Q)\setminus \{a\}$. If $|A|\ge 3$, then $A'\cup \{a,b\}$ is $K_{5}$-admissible by Claim 2.11, where $A'$ is any 3-element subset of *A*. If $|A|\le 2$, then $|V(Q)\setminus (A\cup \{a\})|\ge 3$, then $A''\cup \{a,b\}$ is $K_{5}$-admissible, where $A''$ is any 3-element subset of $V(Q)\setminus (A\cup \{a\})$. $\square$

Now we are ready to conclude the proof of Lemma 2.8, and therefore the proof of Lemma 2.7.

#### Proof of Lemma 2.8

The choice $k_{1}=\lceil {1}/{\epsilon _{3}}\rceil +1$ suffices. Indeed, we proved that if (*R*, *w*) does not contain an (*H*, 0)-admissible subgraph for any $H\in \mathscr {H}$, then $\phi (Q)\ge {1}/{4}$. But, by (1), we have

$$\begin{aligned} w(R)=\frac{k^{2}}{2}\biggl (\phi (Q)-\frac{1}{k}\biggr )>\frac{k^{2}}{2}\biggl (\frac{1}{4}-\epsilon _{3}\biggr ), \end{aligned}$$

finishing the proof. $\square$

### Proof of Theorem 2.4

In this section we put everything together to finish the proof of Theorem 2.4.

#### Proof of Theorem 2.4

Let $\mathscr {H}$ be the family of partial subdivisions of $K_{5}$ with at most eight vertices. Let *C* be the constant given by Lemma 2.1. Let $\epsilon _{2}=\epsilon$, $\epsilon _{1}=\min {\{{C}/{2},\epsilon _{1}(\epsilon _{2})\}}$, and $k_{0}=k_{0}(\epsilon _{2})$, where $\epsilon _{1}(\epsilon _{2})$ and $k_{0}(\epsilon _{2})$ are the constants given by Lemma 2.7. Set $\beta =C$ and $\lambda ={\beta }({\epsilon _{1}}/{2})^{128}/({8h})$. By the regularity lemma, there exists $M=M(k_{0},\lambda )$ such that *G* has a $\lambda$-regular partition $(V_{1},\dots ,V_{k})$, where $k_{0}\le k\le M$. Let (*R*, *w*) be the corresponding reduced graph. Then $$|E(R)|\ge \left( {\begin{array}{c}k\\ 2\end{array}}\right) -\lambda k^{2}> {k^{2}}(1-\epsilon _{1})/{2}.$$ Also,

$$\frac{n^{2}}{k^{2}}w(R)\le |E(G)|\le \biggl (\frac{1}{4}-\epsilon _{2}\biggr )\frac{n^{2}}{2},$$

so $w(R)\le {k^{2}}({1}/{4}-\epsilon _{2})/{2}$. But then, by Lemma 2.7, (*R*, *w*) contains an $(H,\epsilon _{1})$-admissible subgraph for some $H\in \mathscr {H}$. Let $h=|V(H)|\le 8$.

Let $\delta =({\epsilon _{1}}/{2})^{128}/({2M})$ and $\alpha =4\delta <({\epsilon _{1}}/{2})^{16}/({2M})$. Then the parameters $h,\alpha ,\beta ,\delta ,\lambda ,\epsilon _{1}$ satisfy the conditions of Lemma 2.5. Hence, if *G* is $(\alpha ,\beta )$-dense and $\delta$-full, it contains a weak 2-subdivision of *H* as an induced subgraph. Note that a weak subdivision of a partial subdivision of $K_{5}$ contains a weak subdivision of $K_{5}$ as an induced subgraph. Thus, *G* contains a weak subdivision of $K_{5}$ as an induced subgraph.$\square$

## Concluding Remarks

It follows from the combination of Theorems 1.3 and 2.4 that if *G* is a graph with *n* vertices and at most ${n^{2}}({1}/{4}-\epsilon )/{2}$ edges such that *G* does not contain a weak subdivision of $K_{5}$ as an induced subgraph, then $\overline{G}$ contains a linear sized bi-clique. It would be interesting to decide whether this statement can be generalized to graphs with no induced weak subdivision of the complete graph $K_{t}$.

### Conjecture 3.1

For every $\epsilon >0$ and integer $t\ge 3$, there exists $\delta >0$ such that the following holds. If *G* is a graph with *n* vertices and at most ${n^{2}}({1}/({t-1})-\epsilon )/{2}$ edges such that *G* does not contain an induced weak subdivision of $K_{t}$, then $\overline{G}$ contains a bi-clique of size at least $\delta n$.

Note that if this conjecture is true, it is sharp. Indeed, if *G* is a graph whose vertex set can be partitioned into $V_{1},\dots , V_{t-1}$ such that $V_{i}$ spans a clique for $i=1,\dots ,t-1$, then *G* does not contain a weak subdivision of $K_{t}$. But using standard probabilistic techniques, it is easy to construct graphs *G* with less than ${n^{2}}({1}/({t-1})+\epsilon )/{2}$ edges and such that the size of the largest bi-clique in $\overline{G}$ is $O((\log n)/{\epsilon })$. Moreover, a positive answer to Conjecture 3.1 would have similar implications as Theorem 1.2 for intersection graphs of curves defined on certain surfaces. We omit the details.

In order the prove Conjecture 3.1, it would be enough to prove the following generalization of Proposition 2.12, as this is the only part of our proof that does not apply to general families of graphs $\mathscr {H}$.

### Conjecture 3.2

Let $\mathscr {H}$ be the family of partial subdivisions of $K_{t}$. If $(Q,\phi )$ has no *H*-admissible subgraph for any $H\in \mathscr {H}$, then $\phi (Q)\ge {1}/({t-1})$.

We can prove the following slightly weaker version of Conjecture 3.1, by proving a slightly weaker version of Conjecture 3.2.

### Theorem 3.3

For every integer $t\ge 3$, there exists $\delta >0$ such that the following holds. If *G* is a graph with *n* vertices and at most $n^{2}/(4(t-1))$ edges which does not contain an induced weak subdivision of $K_{t}$, then $\overline{G}$ contains a bi-clique of size at least $\delta n$.

### Proof (sketch)

It is enough to show that there exists $\epsilon >0$ such that if $(Q,\phi )$ has no $K_{t}$-admissible subgraph, then $\phi (Q)\ge {1}/({2(t-1))}+\epsilon$. Let $(Q,\phi )$ be the vertex-weighted graph on vertex set [*s*] with no $K_{t}$-admissible subgraph. Then $s< R(t)$, where *R*(*t*) is the usual Ramsey number, that is, *R*(*t*) is the smallest integer *N* such that every graph on *N* vertices contains a clique or an independent set of size *t*. Indeed, if $s\ge R(t)$, then *Q* contains a clique or an independent set of size *t*, but both are $K_{t}$-admissible. Now we show that if

$$\begin{aligned} \phi (Q)\ge \frac{1}{2s}+\frac{1}{2(t-1)}, \end{aligned}$$

then *Q* contains an independent set of size *t*, which is impossible. But then setting $\epsilon ={1}/({2R(t)})$ finishes the proof.

By a similar Turán-type argument as in Proposition 2.10, one can prove the following: given *s*, *t*, and a weight function $\phi :[s]\rightarrow [0,1]$, if *Q* is the graph on vertex set [*s*] that minimizes $\phi (Q)$ while having independence number at most $t-1$, then *Q* has the following form. The vertex set of *Q* can be partitioned into $t-1$ parts $V_{1},\dots ,V_{t-1}$ such that $V_{i}$ induces a clique in *Q* for $i=1,\dots ,t-1$. But then

$$\begin{aligned} \phi (Q)&=\sum _{i=1}^{s}\phi (i)^{2}+\sum _{i=1}^{t-1}\sum _{a,b\in V_{i},a<b}\!\!\phi (a)\phi (b)\\&\qquad \qquad =\frac{1}{2}\sum _{i=1}^{s}\phi (i)^{2}+\frac{1}{2}\sum _{i=1}^{t-1}\Biggl (\sum _{a\in V_{i}}\phi (a)\Biggr )^{\!2}\ge \frac{1}{2s}+\frac{1}{2(t-1)}. \end{aligned}$$

[-36pt] $$\square $$

## Footnotes

1 It is easy to show that for every family of convex sets, there is a family of *x*-monotone curves with the same intersection graphs.

2 The family of cographs is the minimal family of graphs that contains the single vertex graph, and closed under taking disjoint unions and complementation. It is well known that a graph is a cograph if and only if it contains no induced path of length 3.
